# miR-23a/b promote tumor growth and suppress apoptosis by targeting PDCD4 in gastric cancer

**DOI:** 10.1038/cddis.2017.447

**Published:** 2017-10-05

**Authors:** Xiuting Hu, Yanbo Wang, Hongwei Liang, Qian Fan, Ruichi Zhu, Jiayi Cui, Weijie Zhang, Ke Zen, Chen-Yu Zhang, Dongxia Hou, Zhen Zhou, Xi Chen

**Affiliations:** 1 State Key Laboratory of Pharmaceutical Biotechnology, School of Life Sciences, Jiangsu Engineering Research Center for MicroRNA Biology and Biotechnology, NJU Advanced Institute for Life Sciences (NAILS), School of Life Sciences, Nanjing University, 163 Xianlin Road, Nanjing 210023, Jiangsu, China; 2 Department of Lymphoma, National Clinical Research Center of Cancer, Key Laboratory of Cancer Prevention and Therapy, Tianjin Medical University Cancer Institute and Hospital, Tianjin 300060, China; 3 Hong Kong University of Science and Technology, Clear Water Bay, Kowloon, Hong Kong, China; 4 Department of Microbiology, Harbin Medical University, Heilongjiang Provincial Key Laboratory for Infection and Immunity, Key Laboratory of Etiology of Heilongjiang Province Education Bureau, Harbin, China; 5 Department of General Surgery, Affiliated Drum Tower Hospital of Nanjing University Medical School, 321 Zhongshan Road, Nanjing, Jiangsu 210008, China

## Abstract

MicroRNAs (miRNAs) are short non-coding RNAs of 21–23 nucleotides that play important roles in virtually all biological pathways in mammals and in other multicellular organisms. miR-23a and miR-23b (miR-23a/b) are critical oncomiRs (miRNAs that are associated with human cancers) of gastric cancer, but their detailed roles in the initiation and progression of gastric cancer remain to be elucidated. In this study, we found that miR-23a/b were consistently upregulated in gastric cancer tissues. We then investigated the molecular mechanisms through which miR-23a/b contribute to gastric cancer and identified programmed cell death 4 (PDCD4) as a direct target gene of miR-23a/b. In contrast to the upregulated expression levels of miR-23a/b, PDCD4 protein levels were dramatically downregulated and inversely correlated with miR-23a/b in gastric cancer tissues. Moreover, we observed that cell apoptosis was increased by miR-23a/b inhibitors and decreased by miR-23a/b mimics in gastric cancer cells and that the restoration of PDCD4 expression attenuated the anti-apoptotic effects of miR-23a/b in gastric cancer cells, indicating that PDCD4 is a direct mediator of miR-23a/b functions. Finally, we showed that miR-23a/b significantly suppressed PDCD4 expression and enhanced tumor growth in a gastric cancer xenograft mouse model. Taken together, this study highlights an important role for miR-23a/b as oncomiRs in gastric cancer through the inhibition of PDCD4 translation. These findings may shed new light on the molecular mechanism of gastric carcinogenesis and provide a new avenue for gastric cancer treatment.

Gastric cancer is the fourth most frequently diagnosed cancer worldwide, which varies widely in different countries and presents the highest occurrence in Eastern Asia.^1^ Although several screening techniques (e.g., gastric endoscopy, barium meal photofluorography and serum pepsinogen) have been proposed as screening methods for the early detection of gastric cancer, most patients are diagnosed at an advanced stage with a dismal outcome.^2^ While some new drugs have been developed for the prevention and treatment of gastric cancer,^3^ most advanced gastric cancer patients continue to suffer a poor prognosis. The exact mechanisms contributing to the origin and development of gastric cancer remain complex and obscure, and it is important to explore the molecular basis of gastric cancer and to identify new therapeutic targets for this disease.

miRNAs are a class of small non-coding RNA molecules (21–23 nucleotides in length) that regulate gene expression at the post-transcriptional level.^4, 5^ miRNAs bind targeted mRNAs at complementary sites in the 3′-untranslated regions (3′-UTRs), thereby inhibiting the translation or favoring the destabilization of mRNAs, which depends on the degree of nucleotide pairing.^6, 7^ Through this mechanism of action, miRNAs regulate diverse cellular functions and play vital roles in a wide variety of physiological and pathological cellular processes.^8^ Importantly, aberrant miRNA expression is observed in various human cancers, including gastric cancer.^9^ Furthermore, anomalous miRNAs can exert an enormous effect by suppressing oncogenes or tumor suppressors, thereby functioning as tumor-suppressive miRNAs or oncogenic miRNAs during carcinogenesis. Among the miRNAs correlated with tumorigenesis, miR-23a and miR-23b (herein referred to as miR-23a/b) are among the most important. miR-23a/b belong to the miR-23~27~24 cluster: miR-23a is located in the miR-23a~27a~24-2 cluster within the 19p13 chromosomal region, whereas miR-23b is located in the miR-23b~27b~24-1 cluster within the 9q22 chromosomal region.^10, 11^ Notably, miR-23a/b are enhanced in acute lymphoblastic leukemia, acute myeloid leukemia, bladder cancer, glioblastoma, pancreatic cancer, uterine leiomyoma, hepatocellular carcinoma and gastric cancer.^12, 13, 14, 15, 16, 17^ However, although several papers regarding the association of miR-23a/b with cancer have been published, the detailed roles of miR-23a/b in the initiation and progression of gastric cancer remains largely unknown. The aim of this study was to evaluate the association of miR-23a/b expression with gastric cancer and to explore the potential novel target genes of miR-23a/b.

In this study, we found that miR-23a/b levels were consistently upregulated in gastric cancer tissues. Subsequently, we showed that miR-23a/b enhanced tumor growth in a gastric cancer xenograft mouse model. Furthermore, we identified potential target genes of miR-23a/b and found that miR-23a/b inhibit the apoptosis of gastric cancer cells by directly targeting an important tumor suppressor, programmed cell death 4 (PDCD4).

## Results

### miR-23a/b are upregulated in gastric cancer tissues

**Figure 1:**
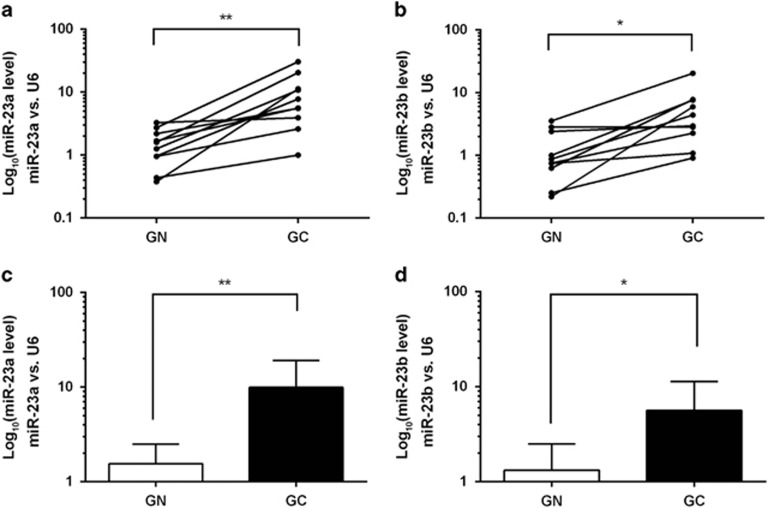
Expression levels of miR-23a/b in gastric cancer tissues. (**a**,**b**) Quantitative RT-PCR analysis of the individual alteration of miR-23a/b in 10 pairs of gastric cancer tissue (GC) compared with matched normal adjacent tissue (GN) samples. (**c**,**d**) Quantitative RT-PCR analysis of the mean expression levels of miR-23a/b in 10 pairs of gastric cancer tissue (GC) and matched normal adjacent tissue (GN) samples. Each bar represents the mean±SD values. (*P<0.05; **P<0.01)

We first determined the expression patterns of miR-23a/b in human gastric cancer tissues. By measuring the expression levels of miR-23a/b in 10 pairs of gastric cancer tissues and normal adjacent tissues with quantitative RT-PCR, we found that miR-23a/b levels were consistently increased in gastric cancer tissues compared with non-cancerous tissues (Figure 1). Moreover, we downloaded the miRNA expression data from The Cancer Genome Atlas (TCGA) website and analyzed the expression profiles of miR-23a/b in 42 normal tissues and 476 gastric cancer tissues. The results indicated again that miR-23a/b were upregulated in gastric cancer tissues (Supplementary Figure 1).

### Prediction of PDCD4 as a target gene of miR-23a/b

**Figure 2:**
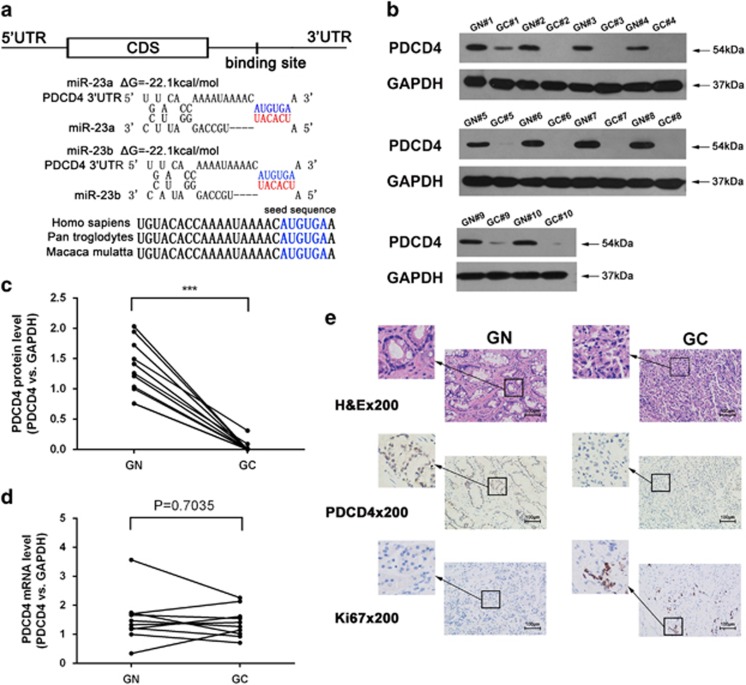
PDCD4 was predicted as a target of miR-23a/b and was downregulated in gastric cancer tissues. (**a**) Schematic description of the hypothetical duplexes formed by the interaction between the binding sites in the PDCD4 3′-UTR (top) and miR-23a/b (bottom). The predicted free energy value of each hybrid is indicated. The seed sequences and seed recognition sites are indicated in red and blue, respectively, and all nucleotides in these regions are highly conserved in several species. (**b**,**c**) Western blotting analysis of PDCD4 protein levels in 10 pairs of GC and GN samples. (**b**) representative image; (**c**) quantitative analysis. (**d**) Quantitative RT-PCR analysis of PDCD4 mRNA levels in the same 10 pairs of GC and GN samples. (**e**) Representative H&E-stained, PDCD4-stained and Ki-67-stained sections of the GC and GN samples. (***P<0.001)

To explore the molecular mechanism by which miR-23a/b contributes to gastric cancer progression, three computational algorithms, TargetScan,^18^ PicTar^19^ and miRanda,^20^ were used in combination to search for potential targets of miR-23a/b. Among the candidates, PDCD4, a potent tumor suppressor gene that is frequently downregulated in human cancers,^21, 22^ was predicted to be a miR-23a/b target by all three of the algorithms and was selected for further experimental verification. The predicted interaction between miR-23a/b and the target site in the PDCD4 3′-UTR is illustrated in Figure 2a. The 3′-UTR of PDCD4 contains one conserved binding site for miR-23a/b. There was perfect base-pairing between the seed region (the seed sequence that encompasses the first 2–8 bases of the mature miRNA 5′ end) and the cognate target. The minimum free energy value of the hybrid was −22.1 kcal/mol, which is well within the range of genuine miRNA-target pairs.

### Detection of inverse correlations between miR-23a/b and PDCD4 protein levels in gastric cancer tissues

Since miRNAs are generally thought to have an opposite expression pattern to that of their targets,^5^ we next investigated whether miR-23a/b expression was inversely correlated with PDCD4 expression in gastric cancer tissues. We measured the expression pattern of PDCD4 in the same 10 pairs of gastric cancer tissues and normal adjacent tissues. PDCD4 protein levels were consistently reduced in gastric cancer tissues (Figures 2b and c). In contrast, PDCD4 mRNA levels did not significantly differ between the cancerous and non-cancerous tissues (Figure 2d), which is in accordance with a post-transcriptional mechanism that is involved in the regulation of PDCD4. Immunohistochemical staining of PDCD4 and Ki-67 in gastric cancer tissues and normal adjacent tissues also revealed the presence of higher proliferative activity (indicated by the staining intensity of Ki-67-positive cells) but lower PDCD4 levels (indicated by the staining intensity of PDCD4-positive cells) in the gastric cancer tissues (Figure 2e).

### Validation of PDCD4 as a direct target of miR-23a/b

**Figure 3:**
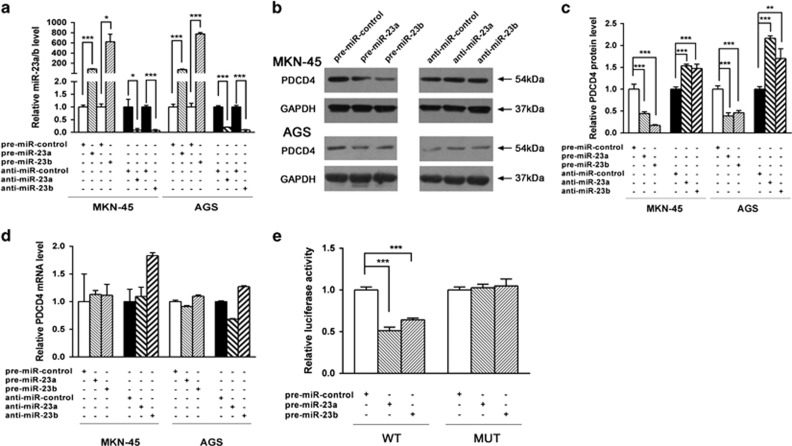
PDCD4 was a direct target of miR-23a/b. (**a**) Quantitative RT-PCR analysis of miR-23a/b levels in MKN-45 and AGS cells transfected with equal doses of pre-miR-23a/b, anti-miR-23a/b or scrambled negative control RNAs (pre-miR-control or anti-miR-control). (**b**,**c**) Western blotting analysis of PDCD4 protein levels in MKN-45 and AGS cells transfected with equal doses of the pre-miR-23a/b, anti-miR-23a/b or scrambled negative control RNAs. (**b**) representative image; (**c**) quantitative analysis. (**d**) Quantitative RT-PCR analysis of PDCD4 mRNA levels in MKN-45 and AGS cells transfected with equal doses of pre-miR-23a/b, anti-miR-23a/b or scrambled negative control RNAs. (**e**) Direct recognition of the PDCD4 3′-UTR by miR-23a/b. Firefly luciferase reporters containing either wild-type (WT) or mutant (MUT) miR-23a/b binding sites in the PDCD4 3′-UTR were co-transfected into HEK293T cells along with equal doses of pre-miR-23a/b or pre-miR-control. The cells were assayed using a luciferase assay kit 24 h post-transfection. Firefly luciferase values were normalized to *β*-gal activity, and the results were calculated as the ratio of firefly luciferase activity in the miR-23a/b-transfected cells normalized to the pre-miR-control-transfected cells. (*P<0.05; **P<0.01; ***P<0.001)

The correlation between miR-23a/b and PDCD4 was further examined by evaluating PDCD4 expression levels in two human gastric cancer cell lines, MKN-45 and AGS, after overexpression or knockdown of miR-23a/b. As anticipated, cellular miR-23a/b levels were significantly increased when MKN-45 and AGS cells were transfected with miR-23a/b mimics and were decreased when treated with miR-23a/b antisenses (Figure 3a). Consequently, the protein levels of PDCD4 were significantly reduced by the introduction of miR-23a/b mimics in MKN-45 and AGS cells, whereas miR-23a/b antisenses significantly increased the PDCD4 protein levels (Figures 3b and c). To determine the level at which miR-23a/b regulate PDCD4 expression, we repeated the above experiments and examined the expression of PDCD4 mRNA after transfection. Overexpression or knockdown of miR-23a/b did not affect the mRNA levels of PDCD4 (Figure 3d). These results demonstrated that miR-23a/b specifically regulate PDCD4 protein expression at the post-transcriptional level, which is the most common mechanism for animal miRNAs.

To confirm that miR-23a/b directly target the presumed binding site in the PDCD4 3′-UTR and negatively regulate PDCD4 expression, a luciferase reporter assay was performed. The PDCD4 3′-UTR containing the presumed miR-23a/b binding site was fused downstream of the firefly luciferase gene in a reporter plasmid. The recombination plasmid was co-transfected into HEK293T cells along with miR-23a/b mimics. As expected, overexpression of miR-23a/b resulted in ~50% reduction of luciferase reporter activity (Figure 3e). Furthermore, we introduced a point mutation into the corresponding complementary site in the PDCD4 3′-UTR to disrupt the predicted miR-23a/b binding site. The mutated luciferase reporter was unaffected by overexpression of miR-23a/b (Figure 3e). This finding indicated that the binding site of PDCD4 strongly contributes to the miRNA-mRNA interaction. In conclusion, our results demonstrated that miR-23a/b directly bind to the 3′-UTR of the PDCD4 transcript to suppress PDCD4 expression.

### miR-23a/b suppress apoptosis in gastric cancer cells by inhibiting PDCD4

**Figure 4:**
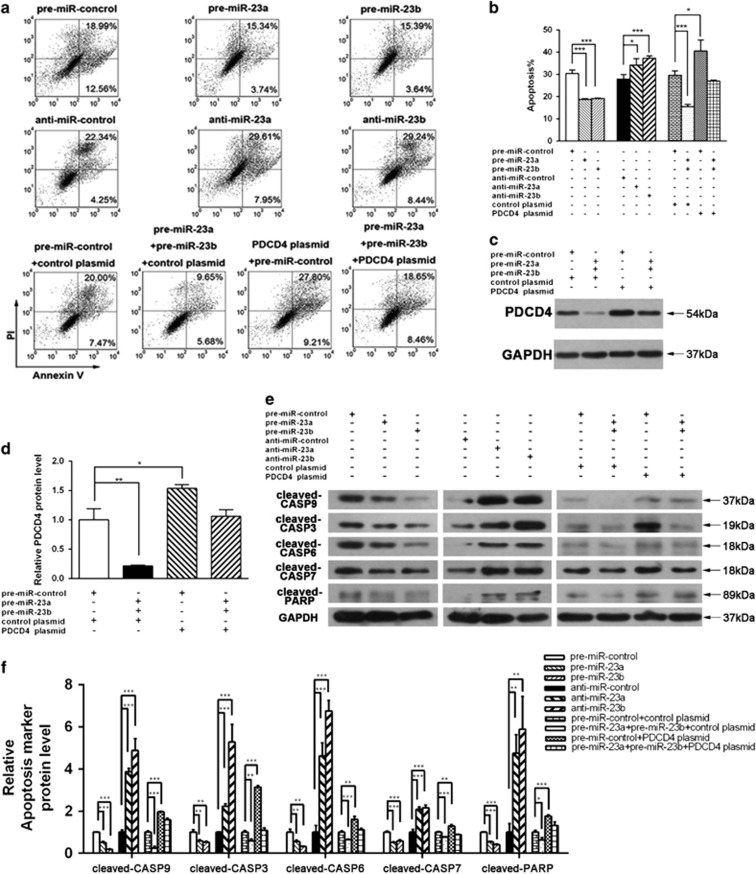
Effects of miR-23a/b and PDCD4 on the apoptosis of gastric cancer cells. (**a**,**b**) Apoptosis assays were performed 24 h after the transfection of MKN-45 cells with equal doses of pre-miR-23a/b, anti-miR-23a/b or scrambled negative control RNAs (pre-miR-control or anti-miR-control), or with equal doses of pre-miR-control plus control plasmid, pre-miR-23a/b plus control plasmid, pre-miR-control plus PDCD4-overexpression plasmid or pre-miR-23a/b plus PDCD4-overexpression plasmid. (**a**) representative image; (**b**) quantitative analysis. (**c,d**) Western blotting analysis of PDCD4 protein levels in MKN-45 cells transfected with equal doses of pre-miR-control plus control plasmid, pre-miR-23a/b plus control plasmid, pre-miR-control plus PDCD4-overexpression plasmid or pre-miR-23a/b plus PDCD4-overexpression plasmid. (**c**) representative image; (**d**) quantitative analysis. (**e**,**f**) Western blotting analysis of the levels of cleaved CASP9, 3, 6, 7 and PARP in MKN-45 cells transfected with equal doses of pre-miR-23a/b, anti-miR-23a/b or scrambled negative control RNAs (pre-miR-control or anti-miR-control), or with equal doses of pre-miR-control plus control plasmid, pre-miR-23a/b plus control plasmid, pre-miR-control plus PDCD4-overexpression plasmid or pre-miR-23a/b plus PDCD4-overexpression plasmid. (**e**) representative image; (**f**) quantitative analysis. (*P<0.05; **P<0.01; ***P<0.001)

Because PDCD4 is a well-known pro-apoptotic gene,^23^ we investigated whether miR-23a/b may suppress gastric cell apoptosis by silencing PDCD4. We first investigated the effects of miR-23a/b on MKN-45 cell apoptosis via flow cytometry. The apoptosis assay showed that the percentage of apoptotic cells was significantly lower in cells transfected with miR-23a/b mimics but was higher in cells transfected with miR-23a/b antisenses (Figures 4a and b). Thus, miR-23a/b function as anti-apoptotic factors in gastric cancer cells. Subsequently, we investigated whether the knockdown or overexpression of PDCD4 would impact apoptosis in MKN-45 cells. To knockdown PDCD4, a siRNA sequence targeting human PDCD4 ORF was designed and transfected into MKN-45 cells. For the overexpression of PDCD4, a plasmid designed to specially express the full-length ORF of PDCD4 without the miR-23a/b-responsive 3′-UTR was constructed and transfected into MKN-45 cells. The efficiency of knockdown or overexpression of PDCD4 is demonstrated in Supplementary Figure 2. As anticipated, transfecting PDCD4 siRNA markedly decreased the percentage of apoptotic cells when compared to the cells transfected with control siRNA, whereas transfecting the PDCD4-overexpression plasmid increased cell apoptosis (Supplementary Figure 3). Thus, the inhibition of cell apoptosis by PDCD4 knockdown was similar to that elicited by miR-23a/b overexpression, further indicating that miR-23a/b and PDCD4 have opposing effects on cell apoptosis.

To investigate if miR-23a/b may regulate the apoptosis of gastric cancer cells through a PDCD4-dependent manner, we co-transfected MKN cells with miR-23a/b mimics and PDCD4-overexpression plasmid. Compared with cells transfected with miR-23a/b mimics plus control plasmid, the cells transfected with miR-23a/b mimics plus PDCD4-overexpression plasmid exhibited a significantly higher PDCD4 protein level (Figures 4c and d), suggesting that the overexpression of miR-23a/b-resistant PDCD4 was sufficient to rescue the suppression of PDCD4 by miR-23a/b. Consequently, MKN-45 cells simultaneously transfected with miR-23a/b mimics and the PDCD4-overexpression plasmid showed significantly higher apoptotic rates than the cells transfected with miR-23a/b mimics plus control plasmid (Figures 4a and b), suggesting that the overexpression of miR-23a/b-resistant PDCD4 was sufficient to attenuate the anti-apoptotic effects of miR-23a/b.

Apoptosis is orchestrated by Caspases, a family of cysteine proteases that cleave their substrates on the carboxy-terminal side of specific aspartic acid residues.^24^ Previous study has shown that introduction of PDCD4 induced the cleavage of CASP9, 3, 6, 7 and PARP in tumor cells.^25^ To further validate that miR-23a/b can suppress gastric cell apoptosis by regulating PDCD4, we investigated the effects of miR-23a/b and PDCD4 on several well-characterized biochemical markers for apoptosis, including cleaved-CASP9, 3, 6, 7 and PARP. As anticipated, transfection of miR-23a/b mimics reduced the cleavage of CASP9, 3, 6, 7 and PARP in MKN-45 cells, whereas transfection of miR-23a/b antisenses induced the cleavage of CASP9, 3, 6, 7 and PARP (Figures 4e and f). When MKN-45 cells were simultaneously transfected with miR-23a/b mimics plus PDCD4-overexpression plasmid, restoration of PDCD4 expression decreased the effects of miR-23a/b-mediated suppression of the cleavage of CASP9, 3, 6, 7 and PARP in MKN-45 cells (Figures 4e and f). In summary, as miR-23a/b and PDCD4 had opposite expression patterns and biological functions in gastric cancer cells, it is quite possible that miR-23a/b suppress apoptosis in gastric cancer cells by silencing PDCD4.

### miR-23a/b function as oncomiRs in gastric cancer

**Figure 5:**
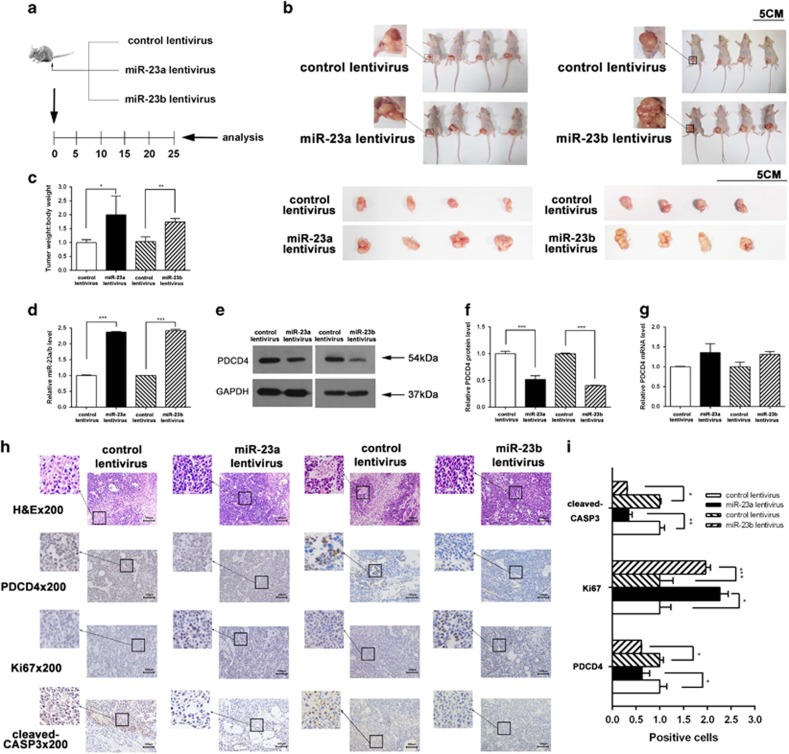
Effects of miR-23a/b on tumor growth in a gastric cancer xenograft mouse model. (**a**) Flow chart of the experimental procedure. MKN-45 cells were infected with a control lentivirus or lentiviruses to overexpress miR-23a or miR-23b. After infection, MKN-45 cells (2 × 10^7^ cells per 0.1 ml) were subcutaneously implanted into 6-week-old SCID mice (8 mice per group), and tumor growth was evaluated on day 25 after cell implantation. (**b**) Representative images of the implanted mice and tumors from the implanted mice. (**c**) Quantitative analysis of the tumor weights. (**d**) Quantitative RT-PCR analysis of miR-23a/b levels in the tumors from implanted mice. (**e**,**f**) Western blotting analysis of PDCD4 protein levels in the tumors from implanted mice. (**e**) representative image; (**f**) quantitative analysis. (**g**) Quantitative RT-PCR analysis of PDCD4 mRNA levels in the tumors from implanted mice. (**h**) Representative H&E-stained, PDCD4-stained, Ki-67-stained and cleaved-CASP3-stained sections of the tumors from implanted mice. (**i**) Quantitative analysis of the sections. (*P<0.05; **P<0.01; ***P<0.001)

We next evaluated the biological effects of miR-23a/b on gastric tumorigenesis in a xenograft mouse model. The human gastric cancer cell line MKN-45 was infected with a control lentivirus or lentiviruses to overexpress miR-23a or miR-23b. The efficient overexpression of miR-23a/b by lentiviral infection is shown in Supplementary Figure 4A. Overexpression of miR-23a/b by lentiviral transfection reduced PDCD4 protein levels in MKN-45 cells (Supplementary Figure 4B and 4C). Then, infected MKN-45 cells were subcutaneously implanted into 6-week-old SCID mice, and tumor growth was evaluated at day 25 after cell implantation (Figure 5a). A significant increase in the size and weight of the tumors was observed in the miR-23a/b-overexpressing group compared to the control group (Figures 5b and c). Subsequently, total RNA was extracted from tumors and used to evaluate the expression levels of miR-23a/b. After 25 days of xenograft growth *in vivo*, tumors from the miR-23a/b-overexpressing groups showed significant increase in miR-23a/b expression compared to tumors from the control group (Figure 5d). Likewise, tumors from the mice infected with miR-23a/b-overexpressing MKN-45 cells displayed reduced PDCD4 protein levels, but not mRNA levels, compared to tumors from the control group (Figures 5e and g). Furthermore, tumor tissues were embedded in paraffin and then stained with H&E for histology examination. The results revealed more cell mitosis in the miR-23a/b-overexpressing groups compared to the control group (Figure 5h). Immunohistochemical staining also revealed the presence of lower levels of PDCD4 in the groups implanted with miR-23a/b-overexpressing cells (Figures 5h and i). Finally, the proliferative activity and apoptosis activity of the tumor cells were assessed via immunohistochemical staining of Ki-67 or cleaved-CASP3, respectively. The tumor cell proliferation rate, as measured by the staining intensity of Ki-67-positive cells, was increased in tumors from the miR-23a/b-overexpressing groups (Figures 5h and i). In contrast, the tumor cell apoptosis rate, as measured by the staining intensity of cleaved-CASP3-positive cells, was decreased in tumors from the miR-23a/b-overexpressing groups (Figures 5h and i).

## Discussion

Recently, miRNAs have been specifically linked to critical developmental pathways, and the dysregulation of many miRNAs has been shown to have functional significance for various human diseases, including cardiovascular disorders, inflammatory diseases, infections, developmental disorders, neurodegenerative diseases and numerous types of cancers.^26^ In the context of cancers, dysregulated miRNAs have been reported to play either a tumor-suppressive or an oncogenic role in regulating tumor cell growth, cell cycle process, migration, angiogenesis and metastasis, depending on the function of their target genes.^27^ The identification of cancer-specific oncomiRs and their expression patterns and target genes is critical for understanding the molecular mechanisms of carcinogenesis and may be important for the identification of novel therapeutic targets and drugs and the development of effective screening and prevention tools.^5, 28, 29^

miR-23a/b are involved in the regulation of a wide variety of cellular processes, including cell differentiation, proliferation, apoptosis, migration and cell-cycle distribution, and also play important roles in several types of human cancers with diverse effects. For example, Zhu *et al.*^30^ reported significant upregulation of miR-23a in gastric adenocarcinoma tissues and demonstrated that miR-23a could target IL6R and promote growth in gastric adenocarcinoma cells. In contrast to miR-23a, miR-23b has a dual role in carcinogenesis and functions as either tumor promoter or tumor suppressor.^31^ In this study, we evaluated the expression patterns of miR-23a/b in gastric cancer tissues and observed that both miR-23a and miR-23b could function as an anti-apoptotic factor in gastric cancer cells. The findings that miR-23a/b are co-upregulated in gastric cancer and have concordant cellular functions allowed us to hypothesize that the miR-23a/b combination might play an important role in gastric carcinogenesis. Therefore, we looked for the target genes of miR-23a/b and identified PDCD4 as a co-target. The results indicated that combined miR-23a/b overexpression may be involved in the progression of gastric cancer through co-targeting tumor suppressor PDCD4 in this malignancy. Consistent with this hypothesis, Li *et al.*^31^ also found that Fas is a co-target of miR-23a/b in thymic lymphoma cells. In a future study, the clinical implications of the combined high expression of miR-23a/b in cancer tissues should be evaluated. Theoretically, co-overexpression of miR-23a/b in cancer tissues may be associated with aggressive tumor progression and poor prognosis, and determining the expression patterns of miR-23a/b may help in elucidating the risk for cancer patients.

Many miRNAs work in conjunction with each other to fine-tune gene expression on a global level. To date, most research on miRNAs has focused on the role of individual miRNA in the regulation of a specific gene. It is important to study the cooperative role of multiple miRNAs, as this insight will give us a whole picture of miRNA regulation within the cell. Studies of the functions of co-expressed miR-23a/b are a promising way to decipher the cooperative effects of multiple miRNAs. Although the reason for the co-expression of miR-23a/b is not fully understood, a possibility is that the simultaneous upregulation or downregulation of miR-23a/b is related to their cooperative effects in regulating specific genes and cellular pathways. This type of regulation is important because when miR-23a/b work in concert to repress a gene, the effect may be more efficient and potent than that by a single miRNA. On the other hand, co-regulation by miR-23a/b may be a fail-proof mode of miRNA regulation to ensure that when one member of the miR-23a/b family is disabled by mutations or dysfunction, the other one is still available to exert its biological function. Cooperation among the miR-23a/b family thus provides an interesting area of study that may change our perception of how miRNAs mediate gene regulation.

Transcription factors (TFs) and miRNAs can jointly regulate target gene expression in the forms of feed-forward loops (FFLs) or feedback loops (FBLs). These regulatory loops serve as important motifs in gene regulatory networks and play critical roles in multiple biological processes and different diseases. Major progress has been made in bioinformatics and experimental study for the TF and miRNA co-regulation in recent years. Previous studies reported that miR-223 and three TFs (C/EBP*α*, NFI-A and E2F1) play critical roles in granulocyte differentiation and the occurrence of acute myelocytic leukemia.^32^ Ma *et al.*^33^ found a positive feedback loop comprised of KLF3 and miR-23a promoting the expression of *β*-like globin genes and the miR-23a/27a/24-2 cluster during erythropoiesis. He *et al.*^34^ revealed a positive feedback loop of PI3K-miR-19a, and MAPK-miR-23b/27b in endothelial cells under shear stress. We also showed that hypermethylated in cancer 1 (HIC1) and miR-23~27~24 clusters form a double-negative feedback loop in breast cancer.^35^ In this study, we predicted transcription factors that can potentially regulate miR-23a/b. Through bioinformatics analysis, we found some signaling pathways and transcription factors that may take part in regulation of miR-23a/b and PDCD4. For example, PDCD4 has been reported to play a role as an inhibitor in AP-1 signaling pathway,^36^ and AP-1 signaling has been shown to activate miR-23a expression.^37^ Thus, AP-1/miR-23a/PDCD4 is likely to form a double-negative (overall positive) feedback loop that contributes to gastric cancer progression. AP-1 downregulation leads to a decrease in miR-23a expression and subsequent PDCD4 activation, which in turn results in the depletion of AP-1 expression.

PDCD4 plays a pivotal tumor-suppression role in the occurrence and development of various cancers. In several types of human tumors, such as breast cancer^38^ and hepatocellular carcinoma,^39^ the levels of PDCD4 were significantly decreased or even disappeared in some tissues. It is generally thought that PDCD4 participates in tumorigenesis through the regulation of apoptosis. For example, Wang *et al.*^40^ confirmed that PDCD4 mediates the sensitivity of gastric cancer cells to apoptosis by suppressing FLIP, a negative regulator of apoptosis. In this study, we observed that PDCD4 was consistently reduced in gastric cancer tissues compared with normal adjacent tissues. We also provided evidence that PDCD4 functions as an essential pro-apoptotic factor in gastric cancer: silencing PDCD4 expression in gastric cancer cells through siRNA inhibits apoptosis, whereas overexpressing PDCD4 has a remarkable effect in promoting cell apoptosis. Interestingly, we identified discordance between PDCD4 protein and mRNA levels in human gastric cancer tissues, suggesting that a post-transcriptional regulatory mechanism is involved in PDCD4 repression. Because miRNA is an important mode of post-transcriptional regulation, we searched for miRNAs that target PDCD4 and validated the direct inhibition of PDCD4 translation through miR-23a/b. Furthermore, because miR-23a/b and PDCD4 had opposing effects on cell apoptosis, it is quite possible that miR-23a/b suppressed PDCD4 expression and consequently inhibited cell apoptosis and promoted tumor growth during gastric cancer progression. Interestingly, we showed that the restoration of PDCD4 expression successfully attenuated the anti-apoptotic effects of miR-23a/b on gastric cancer cells, indicating that the targeting of PDCD4 may be a major mechanism through which miR-23a/b exerted its anti-apoptotic function. PDCD4, as a potent tumor suppressor, may serve as a potential new therapeutic target for gastric cancer, but technical limitations make it difficult to stably express PDCD4 *in vivo*. Because miR-23a/b are upstream regulators of PDCD4 and can specifically block the targeted PDCD4 mRNA with high efficiency and convenience, it is possible to downregulate miR-23a/b for restoration of PDCD4 expression *in vivo*. Currently, the correction of cellular miRNA levels has emerged as a potential therapeutic strategy for a broad range of diseases from genetic disorders to cancer and viral infection.^41, 42^ Overexpression of miRNAs can be silenced using antagomirs, and re-expression of miRNAs that are lost in diseases can be achieved by the overexpression of miRNA mimics.^43, 44^ For example, some scientists have already established the potential usefulness of miRNAs as therapeutic molecules against cancers, including the inhibition of cancer cell proliferation by miR-26a in a mouse model of hepatocellular carcinoma^45^ and the prevention of metastasis formation by silencing miR-10b.^46^ With improvements in nanocarrier technology, miRNA-based therapeutics leads to a greater number of medical breakthroughs and has the potential to generate a revolution in the treatment of diseases.^47, 48^ Currently, delivery system utilizing liposomes, cationic polymer nanoparticles, synthetic chemical agents and native RNA transport service (e.g., exosome) are under active development.^49^ Further studies should be performed to characterize the feasibility of targeting miR-23a/b in gastric cancer therapy and to develop simplified and cost-effective delivery system.

In summary, this study not only uncovered the critical roles of miR-23a/b as oncomiRs in gastric cancer but also explored the molecular mechanisms through which miR-23a/b contributed to gastric cancer progression and identified PDCD4 as a direct target gene. Regulation of PDCD4 by miR-23a/b may explain why the upregulation of miR-23a/b during gastric carcinogenesis promotes tumor growth. This study may provide insight into the molecular mechanism of gastric carcinogenesis and open a new avenue for gastric cancer treatment.

## Materials and methods

### Human tissues and cell lines

A total of 10 pairs of gastric cancer tissues and normal adjacent tissues were derived from patients undergoing surgical procedures at the Affiliated Drum Tower Hospital of Nanjing University Medical School (Nanjing, China). All of the patients provided written consent and the Ethics Committee of Nanjing University approved all aspects of this study. Tissue fragments were immediately frozen in liquid nitrogen at the time of surgery and stored at −80 °C. The clinical features of the patients are listed in Supplementary Table 1. The human gastric cancer cell lines MKN-45 and AGS were purchased from the Shanghai Institute of Cell Biology, Chinese Academy of Sciences (Shanghai, China). The cells were cultured in RPMI 1640 medium (Invitrogen, Carlsbad, CA, USA) supplemented with 10% fetal bovine serum (FBS, Gibco, Carlsbad, CA, USA) in a 5% CO_2_ water-saturated atmosphere.

### RNA extraction and quantitative RT-PCR

Total RNA was extracted from the human tissues and culture cells using Trizol reagent (Sigma, St. Louis, MO, USA) according to the manufacturer’s instructions. The total RNA concentration was determined using a BioPhotometer (Eppendorf, Germany). Assays for the quantification of the miRNAs were performed using TaqMan miRNA probes (Applied Biosystems, Foster City, CA, USA) according to the manufacturer’s instructions. Briefly, 1 *μ*g of total RNA was reverse-transcribed to cDNA using AMV reverse transcriptase (TaKaRa, Dalian, China) and a stem-loop RT primer (Applied Biosystems). The reaction conditions were as follows: 16 °C for 30 min, 42 °C for 30 min and 85 °C for 5 min. Real-time PCR was performed using a TaqMan PCR kit in an Applied Biosystems 7300 Sequence Detection System (Applied Biosystems). The reactions were incubated in a 96-well optical plate at 95 °C for 10 min, followed by 40 cycles of 95 °C for 15 s and 60 °C for 1 min. All of the reactions were run in triplicate. After the reaction, the cycle threshold (C_T_) data were determined using fixed threshold settings and the mean C_T_ was determined from the triplicate PCRs. A comparative C_T_ method was used to determine the relative levels of miRNAs. The amount of miRNA relative to the internal control U6 was calculated using the equation 2^−△△CT^, in which △△C_T_=(C_T miR-23a/b_–C_T U6_)_tumor_–(C_T miR-23a/b_–C_T U6_)_control_.

To quantify PDCD4 mRNA, 1* μ*g of total RNA was reverse-transcribed to cDNA using oligo dT and AMV reverse transcriptase (TaKaRa, Dalian, China) in the reaction, which was performed using the following conditions: 42 °C for 60 min and 70 °C for 10 min. Next, real-time PCR was performed with the RT product, SYBER Green Dye (Invitrogen, Carlsbad, CA, USA) and specific primers for PDCD4 and GAPDH. The sequences of the primers were as follows: PDCD4 (sense): 5′-GTTGGCAGTATCCTTAGCATTGG-3′ PDCD4 (antisense): 5′-TCCACATCAGTTGTGCTCATTAC-3′ GAPDH (sense): 5′-GATATTGTTGCCATCAATGAC-3′ and GAPDH (antisense): 5′-TTGATTTTGGAGGGATCTCG-3′. The reactions were incubated at 95 °C for 5 min, followed by 40 cycles of 95 °C for 30 s, 60 °C for 30 s and 72 °C for 30 s. After the reactions were complete, the C_T_ values were determined by setting a fixed threshold. The relative amount of PDCD4 mRNA was normalized to GAPDH.

### Protein extraction and western blotting

Total protein was extracted from the human tissues and culture cells using the RIPA lysis buffer (Beyotime, Shanghai, China) according to the manufacturer’s instructions. The supernatant was collected, and the protein concentration was calculated with a Pierce BCA protein assay kit (Thermo Scientific, Rockford, IL, USA). Then, equivalent quantities of protein were separated by 10% SDS–PAGE and transferred to a PVDF membrane (Millipore, Bedford, MA, USA). After blocking with 5% non-fat milk at room temperature for 1 h, the membranes were immunostained with a primary antibody at 4 °C overnight, washed three times in TBST, and then incubated with a secondary antibody at room temperature for 1 h. The band signals were detected with an enhanced chemiluminescence reagent (Cell Signaling Technology Inc., USA). The protein levels were normalized by probing the same blots with a GAPDH antibody. The primary antibodies were purchased from the following sources: anti-PDCD4 (Santa Cruz, CA, USA); anti-GAPDH (Santa Cruz, CA, USA); anti-cleaved-CASP9 (Cell Signaling Technology Inc., USA); anti-cleaved-CASP3 (Cell Signaling Technology Inc., USA); anti-cleaved-CASP6 (Cell Signaling Technology Inc., USA); anti-cleaved-CASP7 (Cell Signaling Technology Inc., USA); anti-cleaved-CASP9 (Cell Signaling Technology Inc., USA); and anti-cleaved-PARP (Cell Signaling Technology Inc., USA). The intensity of each band was scanned and quantified using imageJ software.

### Overexpression or knockdown of miR-23a/b

Overexpression of miR-23a/b was achieved by transfecting gastric cancer cells with miRNA mimics (synthetic RNA oligonucleotides mimicking precursors of miR-23a/b). Knockdown of miR-23a/b was achieved by transfecting with miRNA inhibitors (chemically modified single-stranded antisense oligonucleotides designed to specifically sequester mature miR-23a/b). Synthetic miR-23a/b mimics (pre-miR-23a/b), inhibitors (anti-miR-23a/b) and scrambled negative control RNAs (pre-miR-control and anti-miR-control) were purchased from GenePharma (Shanghai, China). MKN-45 and AGS cells were seeded in 6-well plates using RPMI 1640 medium supplemented with 10% FBS. The cells were transfected with Lipofectamine 2000 (Invitrogen, Carlsbad, CA, USA) using Opti-MEM Reduced Serum Medium (Gibco, Carlsbad, CA, USA) on the following day when the cells were ~60–70% confluent. For each well, equal amounts of pre-miR-23a/b, pre-miR-control, anti-miR-23a/b or anti-miR-control were used. After 6 h, the medium was changed to RPMI 1640 supplemented with 2% FBS. The cells were collected at 24 or 48 h after transfection and subjected to analysis by quantitative RT-PCR or western blotting.

### SiRNA interference and plasmid construction

SiRNA sequence targeting the human PDCD4 cDNA was designed and synthesized by GenePharma. The siRNA sequence was as follows: 5′-GCGGAAAUGUUAAGAGAUU-3′. A scrambled siRNA was synthesized as a negative control. A mammalian expression plasmid encoding the full-length human PDCD4 open reading frame (ORF) without the miR-23a/b-responsive 3′-UTR was purchased from Invitrogen. An empty plasmid was used as the negative control. The PDCD4 siRNA and the PDCD4 expression plasmid were transfected into MKN-45 cells using Lipofectamine 2000 (Invitrogen, Carlsbad, CA, USA) according to the manufacturer’s instructions. Total RNA and protein were isolated 24 h post-transfection. The PDCD4 mRNA and protein expression levels were assessed by quantitative RT-PCR and western blotting, respectively.

### Luciferase reporter assays

The entire 3′-UTR of the human PDCD4 transcript was amplified by PCR using human genomic DNA as a template. The PCR products were inserted into the p-MIR-reporter plasmid (Ambion, Austin, TX, USA). The insertion was confirmed as correct by sequencing. To test the binding specificity, the sequences that interact with the miR-23a/b seed sequence were mutated, and the mutant PDCD4 3′-UTR was inserted into an equivalent luciferase reporter. For the luciferase reporter assay, HEK293T cells were cultured in 24-well plates. Each well was transfected with 0.2 *μ*g of firefly luciferase reporter plasmid, 0.1* μ*g of a *β*-galactosidase (*β*-gal) expression plasmid (Ambion), and equal amounts 100 pmol of pre-miR-23a/b, anti-miR-23a/b or the scrambled negative control RNAs using Lipofectamine 2000 (Invitrogen, Carlsbad, CA, USA). The *β*-gal plasmid was used as a transfection control. The cells were assayed using a luciferase assay kit (Promega, Madison, WI, USA) at 24 h post transfection.

### Cell apoptosis assays

The apoptosis of MKN-45 cells was examined using an Annexin V-FITC/propidium iodide (PI) staining assay. MKN-45 cells were cultured in 12-well plates and transfected with pre-miR-23a/b, anti-miR-23a/b, PDCD4 siRNA or PDCD4-overexpression plasmid. Pre-miR-control, anti-miR-control, control siRNA and control plasmids served as negative controls. The cells were cultured in serum-depleted medium to induce apoptosis. After 24 h, the floating cells were discarded, and the attached cells were collected. The apoptotic cells were identified through flow cytometry using an Annexin V-FITC/PI staining kit (BD Biosciences, CA, USA). After washing with cold PBS, the cells were re-suspended in binding buffer (100 mM HEPES, pH 7.4, 100 mM NaCl, and 25 mM CaCl_2_), followed by staining with Annexin V-FITC/PI at room temperature for 15 min in the dark. Apoptotic cells were subsequently evaluated by gating PI and Annexin V-positive cells on a fluorescence-activated cell-sorting (FACS) flow cytometer (BD Biosciences, San Jose, CA). All experiments were performed in triplicate.

### Establishment of tumor xenografts in mice

Six-week-old male SCID (severe combined immune deficiency) mice (*nu/nu*) were purchased from the Model Animal Research Center of Nanjing University (Nanjing, China) and were maintained under specific pathogen-free conditions at Nanjing University. MKN-45 cells were infected with a control lentivirus or lentiviruses to overexpress miR-23a or miR-23b. After infection and transfection, MKN-45 cells were subcutaneously injected into xenograft mice (2 × 10^7^ cells per mouse, 8 mice per group). After 25 days, the mice were killed. The tumor xenografts were removed, and the weight of the tumors was measured. Parts of the tumors were used for protein and total RNA extraction, and the remaining tumors were fixed in 4% paraformaldehyde for 24 h and then processed for hematoxylin and eosin (H&E) staining or immunohistochemical staining for PDCD4, Ki-67 and cleaved-CASP3.

### Statistical analysis

All images of western blotting and cell apoptosis assays are representatives of at least three independent experiments. Quantitative RT-PCR and luciferase reporter assays were performed in triplicate, and each experiment was repeated three to five times. The data shown are the mean±SD of at least three independent experiments. The differences were considered statistically significant at *P*<0.05 using Student’s *t*-test. Prism 5.0 software (GraphPad, Inc., La Jolla, CA, USA) was used for data analyzes.

## Publisher’s Note:

Springer Nature remains neutral with regard to jurisdictional claims in published maps and institutional affiliations.
