# Supplementary material for: miR-23a/b promote tumor growth and suppress apoptosis by targeting PDCD4 in gastric cancer
Source: Cell Death Dis. 2017 Oct 5;8(10):e3059–. doi: 10.1038/cddis.2017.447 (PMC5680570; doi:10.1038/cddis.2017.447)
Supplement: Supplementary Material [file cddis2017447x1.doc]

**miR-23a/b promote tumor growth and suppress apoptosis by targeting PDCD4 in gastric cancer**

**Author affiliation:**

Xiuting Hu^1,*^, Yanbo Wang^1,*^, Hongwei Liang^1,*^, Qian Fan^2,*^, Ruichi Zhu^3^, Jiayi Cui^4^, Weijie Zhang^5^, Ke Zen^1^, Chen-Yu Zhang^1^, Dongxia Hou^1,#^, Zhen Zhou^1,#^, Xi Chen^1,#^

**Summary**

The supplementary information includes supplementary tables, figures and figure legends.

**Supplementary Table 1. Clinical features of gastric cancer patients.**

|  | **Age** | **Gender** | **Tumor subtype** | **Pathological Stage** |
| --- | --- | --- | --- | --- |
| **Case #1** | **61** | **male** | **Adenocarcinoma** | **ⅢA** |
| **Case #2** | **63** | **male** | **Adenocarcinoma** | **ⅠB** |
| **Case #3** | **58** | **male** | **Adenocarcinoma** | **ⅡB** |
| **Case #4** | **64** | **male** | **Adenocarcinoma** | **ⅢA** |
| **Case #5** | **56** | **male** | **Adenocarcinoma** | **ⅢC** |
| **Case #6** | **65** | **male** | **Adenocarcinoma** | **ⅡB** |
| **Case #7** | **71** | **female** | **Adenocarcinoma** | **ⅢC** |
| **Case #8** | **65** | **female** | **Adenocarcinoma** | **ⅢB** |
| **Case #9** | **53** | **male** | **Adenocarcinoma** | **Ⅳ** |
| **Case#10** | **70** | **female** | **Adenocarcinoma** | **ⅡA** |

**Supplementary Figure 1. Expression levels of miR-23a/b in gastric cancer tissues. (A)** Relative expression levels of miR-23a in 42 normal tissues and 476 gastric cancer tissues in The Cancer Genome Atlas (TCGA). **(B)** Relative expression levels of miR-23b in 42 normal tissues and 476 gastric cancer tissues in The Cancer Genome Atlas (TCGA). (*** p < 0.001)

**Supplementary Figure 2. Evaluation of the knockdown and overexpression efficiency of PDCD4 in MKN-45 cells. (A)** Quantitative RT-PCR analysis of PDCD4 mRNA levels in MKN-45 cells transfected with PDCD4 siRNAs or the scrambled negative control siRNA, or transfected with the PDCD4 overexpression plasmid or control plasmid. **(B and C)** Western blotting analysis of the PDCD4 protein levels in MKN-45 cells transfected with PDCD4 siRNAs or the scrambled negative control siRNA, or transfected with the PDCD4 overexpression plasmid or control plasmid. B: representative image; C: quantitative analysis. (* p < 0.05; *** p < 0.001)

**Supplementary Figure 3. Effect of PDCD4 siRNA and PDCD4 plasmid on the apoptosis of gastric cancer cells.** The apoptosis assay was performed 24 hours after the transfection of MKN-45 cells with equal doses of control siRNA, PDCD4 siRNA, control plasmid, PDCD4 overexpression plasmid. A: representative image; B: quantitative analysis. (** p < 0.01,*** p < 0.001)

**Supplementary Figure 4. Expression levels of miR-23a/b or PDCD4 protein in MKN-45 cells after transfection with lentiviruses to overexpress miR-23a or miR-23b. (A)**Quantitative RT-PCR analysis of miR-23a/b levels in MKN-45 cells transfected with a control lentivirus or lentiviruses to overexpress miR-23a or miR-23b; **(B and C)** Western blotting analysis of the PDCD4 protein levels in MKN-45 cells transfected with a control lentivirus or lentiviruses to overexpress miR-23a or miR-23b. B: representative image; C: quantitative analysis. (* p < 0.05; *** p < 0.001)

**Supplementary Figures**

**Supplementary Figure 1**


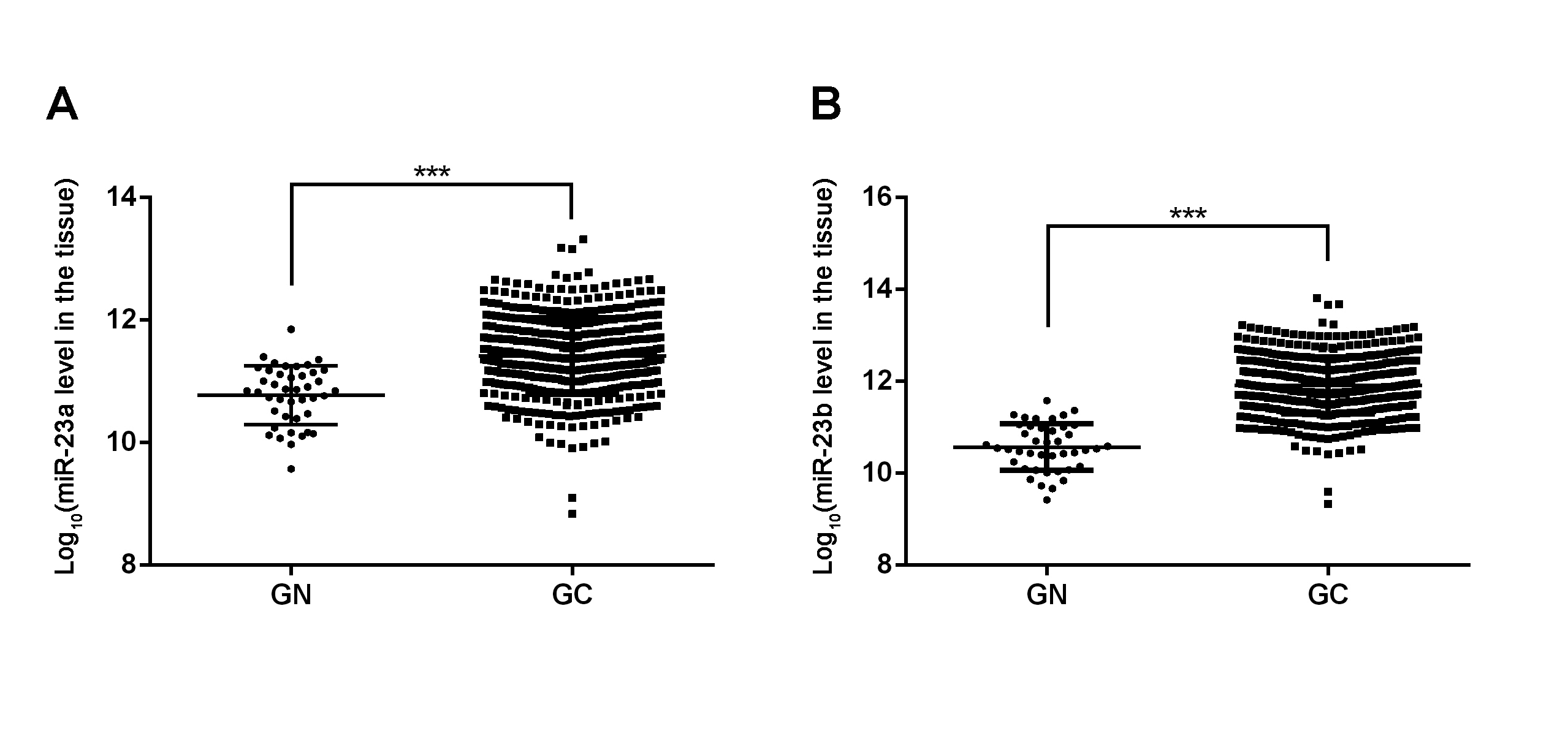


**Supplementary Figure 2**


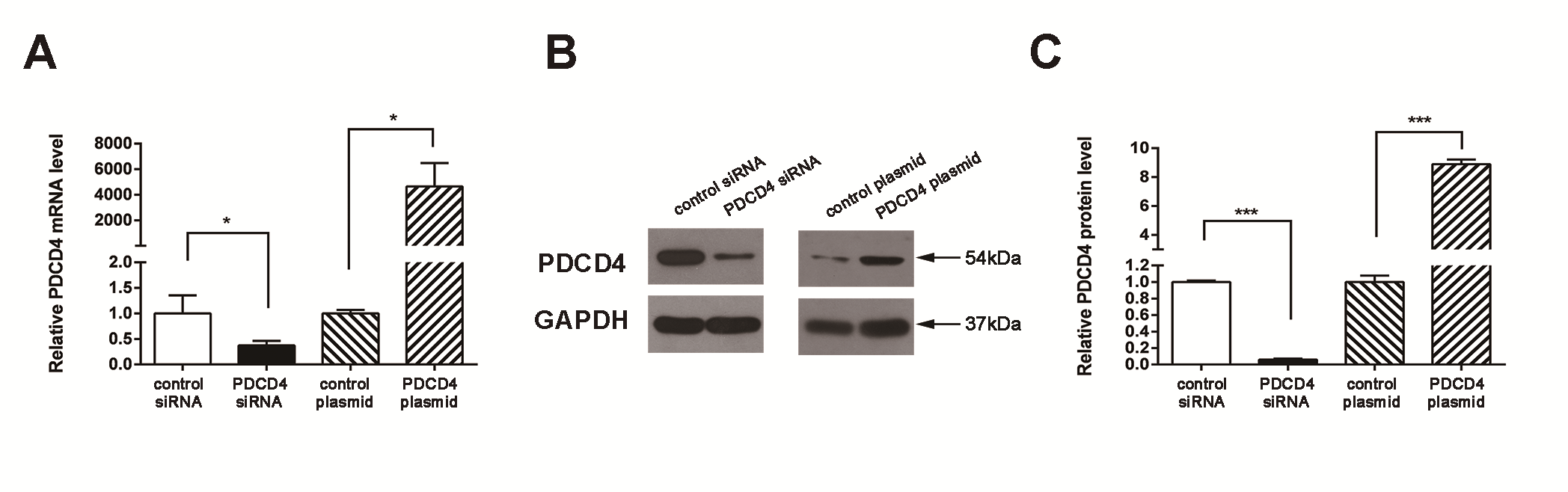


**Supplementary Figure 3**


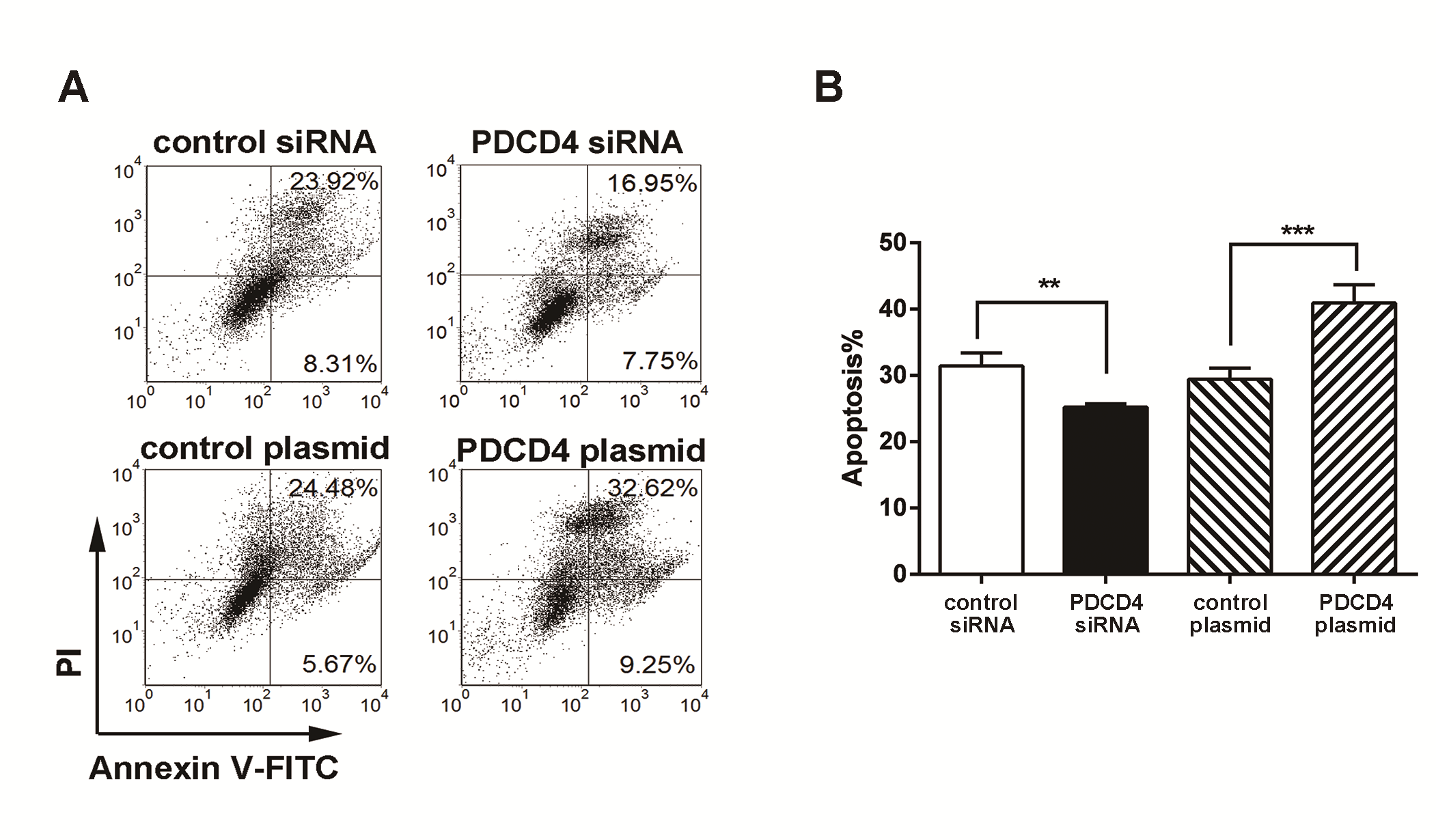
**Supplementary Figure 4**

**
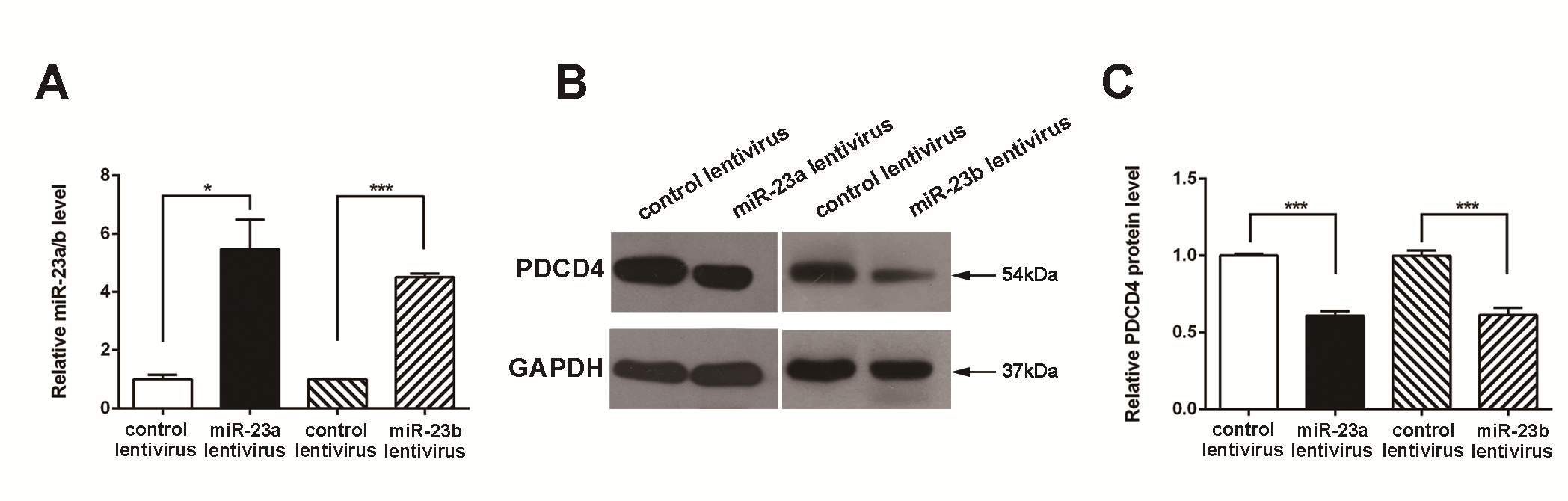
**

Supplementary information is available at ***Cell Death & Disease***’s website.
